# Does digital economy strengthen the income distribution effect of fiscal expenditure? Evidence from China

**DOI:** 10.1371/journal.pone.0290041

**Published:** 2023-08-10

**Authors:** Jie Yan, Xunhua Tu, Jing Zheng

**Affiliations:** 1 School of Public Finance and Taxation, Southwestern University of Finance and Economics, Chengdu, Sichuan, China; 2 Department of Economics & Management, Sichuan Engineering Technical College, Deyang, Sichuan, China; Cavendish University / Kyambogo University, UGANDA

## Abstract

The exponential growth of China’s digital economy has exerted a profound influence on economic advancement and income distribution. To effectively tackle income inequality, it is essential to incorporate the analysis of digital economy development within the framework of fiscal expenditure. This study utilizes a comprehensive panel dataset encompassing 276 cities in China during the period from 2011 to 2020. Employing the fixed-effect model and instrumental variable method, the research investigates the influence of fiscal expenditure on the income gap while investigating the moderating effect of the digital economy. The key findings of the study can be summarized as follows: (1) In general, fiscal expenditure demonstrates a propensity to reduce the income gap. (2) Different categories of fiscal expenditure exhibit distinct effects on the income gap. Social security and employment expenditures do not significantly alleviate the income gap. Conversely, education expenditures and health expenditures tend to exacerbate the income gap. On the other hand, expenditures in agriculture, forestry, and water resources, as well as urban and rural affairs, effectively narrow the income gap. (3) The development of the digital economy enhances the capacity of fiscal expenditure to adjust income distribution, showcasing non-linear effects. From a fiscal expenditure classification perspective, the digital economy primarily enhances the effectiveness of income distribution adjustment for expenditures in sectors such as agriculture, forestry, water resources, and others. Based on these findings, this study proposes a set of future measures aimed at facilitating China’s efforts to reduce the income gap within the framework of the digital economy. These measures encompass expediting the integration of the digital economy with government governance and advocating for the widespread adoption of digital government affairs platforms. By implementing these measures, China can gain valuable insights into effectively addressing income inequality and promoting more equitable economic outcomes within the context of the digital economy.

## Introduction

In recent decades, China has undergone rapid economic growth and transformation. However, this growth has been accompanied by an increase in regional economic disparities and a widening income gap. Despite experiencing fluctuations and a decline from its peak of 0.491 in 2008, the Gini coefficient, a measure of income inequality among residents in China, remains persistently elevated, measuring 0.468 in 2020. Furthermore, there exists a persistent urban-rural divide, with the income ratio between urban and rural residents reaching 2.5 in 2021. This indicates ongoing disparities between these two groups. Similarly, significant regional disparities persist, with notable variations in per capita disposable income among provinces. Based on the per capita disposable income data of 31 provinces in 2021, the income ratio between the highest and lowest income provinces was 3.54 (Shanghai vs. Gansu). The regional income Theil index reflects substantial income differentiation within regions, with an average value of 0.16 in 2011 and peaking at 0.6237, followed by an average value of 0.13 in 2020 with a peak of 0.7868. Despite the significant achievements in poverty alleviation during the 13th Five-Year Plan period, where millions of people were lifted out of poverty, China continues to grapple with considerable income and wealth disparities. The goal of achieving common prosperity remains a formidable challenge that demands concerted efforts and long-term commitment.

In recent years, China’s rapid development of the digital economy has led to its pervasive influence across diverse industries. From 2015 to 2021, the size of the digital economy has escalated from 18.6 trillion yuan to surpass 45 trillion yuan, representing a surge from 27.5% to 39.8% of the GDP. The flourishing digital economy has had extensive and profound repercussions on economic growth, augmenting the overall economic landscape. Concurrently, the digital economy is closely intertwined with income distribution, influencing resource allocation. Against the backdrop of this new digital economy context, it becomes crucial to investigate whether there are novel dynamics in the impact of fiscal expenditure on income distribution adjustment. Such exploration is vital for the continued optimization of fiscal policy.

Throughout the years, the correlation between fiscal expenditure and income inequality has garnered significant scholarly attention. Researchers have conducted numerous studies from various perspectives, leading to diverse conclusions. Some scholars argue that fiscal spending does not effectively address income disparity [1, 2], with limited impact on wealth inequality [3]. In contrast, proponents of an alternative viewpoint posit that fiscal expenditure positively influences income disparity, contributing to the overall reduction of income inequality [4–6]. Specifically, increased investment spending is believed to enhance short-term income distribution patterns [7, 8], while transfer spending can effectively mitigate income inequality among residents [9, 10]. Furthermore, fiscal initiatives aimed at addressing livelihood concerns play a crucial role in shaping income disparity between urban and rural populations. To further optimize the national income distribution pattern, China should consider increasing fiscal expenditure on social security and social welfare programs that reflect livelihood finance [11].

Previous studies conducted by multiple scholars have primarily examined the direct impact of the digital economy on income distribution. These investigations have explored its impact on various determinants and its association with income inequality [12, 13]. However, it is crucial to acknowledge that the influence of the digital economy extends beyond income distribution and also encompasses fiscal expenditure, driven by government digitalization initiatives. The adoption of digital technologies in government operations enhances fiscal transparency, contributing to the reduction of rent-seeking behavior and corruption [14–17]. Consequently, these dynamics have implications for the efficiency of fiscal spending and influence the moderating effect of fiscal expenditure on income inequality [18].

The existing scholarly literature offers limited insights into the intricate relationship among the digital economy, fiscal expenditure, and income inequality. Given the rapid advancement of the digital economy, this study aims to fill this gap by addressing two novel research inquiries. Firstly, it is crucial to explore whether fiscal expenditure amplifies or mitigates the effects of income redistribution, considering the potential of the digital economy to exacerbate disparities in primary income distribution. Secondly, an examination is warranted to determine whether the development of the digital economy strengthens or hampers the capacity of fiscal expenditure to regulate income distribution. By addressing these inquiries, this study not only contributes to resolving income distribution challenges in the current landscape but also provides valuable insights for promoting fairness in income distribution. Furthermore, this study serves as a valuable addition to the existing literature in this field.

This study builds upon the existing literature and focuses on three key aspects using a comprehensive panel dataset encompassing 276 cities in China from 2011 to 2020. Firstly, employing a fixed-effect model and instrumental variable method, the paper examines the moderating effect of fiscal expenditure on the income gap. Moreover, it investigates the potential enhancement of this moderating effect through the digital economy. Secondly, from the standpoint of categorizing fiscal expenditure, the study analyzes the adjusting impact of various types of fiscal expenditure on the income gap, alongside investigating the influence of the digital economy on the adjustment capacity of income disparity within these categorized fiscal expenditures. The primary objective is to provide empirical evidence that informs the formulation and implementation of policies. Thirdly, in measuring the regional income gap, the paper utilizes the Thiel index, which calculates income disparity within a region based on income and population data from districts and counties under the jurisdiction of prefecture-level cities. This methodology allows for a more precise measurement of income disparity among districts and counties within a specific region, in contrast to the conventional use of the Theil index that solely relies on income data from urban and rural residents.

## Theoretical analysis and research hypotheses

### The influence of fiscal expenditure on the income inequality

When considering the association between fiscal expenditure and income distribution, it is crucial to acknowledge the dual impact of fiscal outlays. Firstly, fiscal expenditure directly affects individuals’ income levels through subsidies and transfer payments. Government transfers and income relief programs targeting the impoverished contribute to reducing inequality and poverty [19]. Tang and Sun (2022) observe that fiscal incentives encourage financial institutions to increase agricultural loans, resulting in a significant reduction in urban-rural income inequality, particularly in underdeveloped regions [20]. Secondly, fiscal expenditure impacts the skill proficiency of the labor force by increasing investments in education and employment training. Increased investment in labor education has been shown to improve academic performance and enhance cognitive and noncognitive abilities among students [21]. Furthermore, fiscal spending also influences employment prospects, thus impacting the initial distribution of income. Government fiscal policies, such as direct and indirect subsidies for research and development (R&D) activities, contribute to enhanced production, technological knowledge, and wage levels [22].

Recognizing the substantial influence of disaggregated fiscal expenditures on income disparity is crucial. Firstly, the allocation of fiscal resources towards education yields a profound influence on income distribution, primarily through the molding of residents’ human capital. Government investment in education not only facilitates the improvement of educational equity and the promotion of human capital formation but also enhances the overall skill level of the labor force. Secondly, healthcare fiscal expenditure plays a pivotal role in shaping the income gap by primarily affecting the population’s health status. Good health is a fundamental requirement for individuals to contribute their human capital, and increased government expenditure on healthcare helps provide improved public services to residents while addressing health inequalities among various demographic groups. Thirdly, social security plays a vital role in income redistribution in China. Theoretically, social security expenditure contributes to regulating income disparity, reducing income inequality, and ensuring social equity. However, challenges like inadequate coverage, uneven development, and imperfect system design within China’s social security framework limit its effectiveness in moderating income disparity [23]. Therefore, practical considerations such as expanding coverage, improving system design, and establishing appropriate protection levels are necessary to fulfill the intended role of social security expenditure in income distribution regulation. Fourthly, productive fiscal expenditures, including infrastructure construction, agriculture, forestry, water conservancy, and transportation, play a substantial role in influencing income distribution by enhancing residents’ access to public services. The development of transportation infrastructure, for instance, facilitates mobility between urban and rural areas, creating improved job opportunities and higher wage levels. Consequently, these factors have a direct impact on the pattern of income distribution. Additionally, investments in rural transportation and infrastructure contribute to reducing income disparities and promoting rural development. Increased infrastructure spending is causally linked to reduced inequality, providing improved access to employment and education, particularly benefiting the bottom 40% of the income distribution [8].

Based on the presented analysis, we put forward the following research hypothesis:

**Hypothesis 1**: **Fiscal expenditure plays a pivotal role in diminishing the income gap, with varying effectiveness observed among different categories of fiscal expenditure**.

### The moderating effect of the digital economy

In addition to analyzing the influence of fiscal expenditure on income disparity, it is crucial to explore the implications of the digital economy’s advancement on the redistributive function of fiscal expenditure, considering its pervasive integration across multiple sectors. As the digital economy continues to penetrate diverse domains, it is essential to assess how this phenomenon influences the ability of fiscal expenditure to effectively regulate income disparity.

The digital economy directly affects income distribution. Economic data demonstrates that it has increased overall income levels and contributed to the growth of per capita GDP. However, it is still uncertain whether the digital economy has worsened income inequality. On one hand, the digital economy exacerbates the unequal distribution of factor income by reducing the proportion of labor income within the total income and further favoring capital [24–26]. On the other hand, it has widened the income gap among workers with varying skill levels [13]. Artificial intelligence technology asymmetrically affects the productivity of various technical sectors and influences the distribution of labor income by inducing shifts in labor positions. This effect results in an average annual increase of 0.75% in the income gap between high-skilled and low-skilled sectors [27]. Thirdly, the digital economy has made significant contributions to reducing gender-based income disparity [12], and the accelerated development of women’s cognitive and general skills has played a crucial role in further narrowing the gender wage gap [28]. Fourthly, the digital economy has the potential to alleviate income disparities. Digital finance advances more rapidly in underdeveloped regions, leading to a significant increase in household income, particularly for low-income groups in rural areas. Consequently, the advancement of the digital economy helps narrow the urban-rural divide [29]. Nonetheless, the influence of Internet popularization on the income gap between urban and rural areas in China follows an "inverted U-shaped" pattern [30]. Lastly, the digital economy could potentially widen the income disparity among diverse groups. Variations in the comprehension and utilization of information technology among these groups will influence their ability to generate wealth and exacerbate inequality [31]. The consequences of the dot-com bubble will continue to contribute to the ongoing growth of inequality [32].

The digital economy can strengthen the mitigating impact of fiscal expenditure on income inequality. First, an advanced digital economy expands public access to information, ensuring increased rights to knowledge, participation, and oversight over fiscal expenditures. This reduction in information asymmetry enhances the efficiency and efficacy of fiscal expenditures in income redistribution. The development of the digital economy serves as a technological enabler for governments to improve fiscal transparency and expand public access to pertinent information. This is mainly due to technological innovations that reduce the cost of storing, analyzing, and transmitting information. Consequently, governments can provide a substantial increase in the quantity of financial information available [14]. Additionally, the progress of the Internet has a positive influence on the level of government budget information disclosure. Countries with higher Internet penetration rates are more likely to have increased information disclosure by central governments [33]. Empirical evidence based on data from China supports this notion, indicating a substantial and positive association between Internet usage and fiscal transparency in urban areas [16]. Enhanced fiscal transparency brings numerous benefits, including debt reduction, improved fiscal performance [34], increased government effectiveness, and enhanced efficiency of government spending [18]. Furthermore, increased fiscal transparency plays a crucial role in curbing corruption [17], reducing fiscal spending in sectors vulnerable to rent-seeking behavior, thereby enhancing fiscal spending efficiency [35]. By improving the efficiency of fiscal spending, the ability to address income disparity among the population is enhanced, as the output effect resulting from efficiency improvements at the same level of fiscal spending increases.

Moreover, the increased level of the digital economy enables more effective public participation and monitoring of fiscal expenditure, which in turn contributes to the reduction of corruption and embezzlement. The alignment facilitates an improved synchronization between fiscal resources and the ever-evolving public needs, thereby bolstering the harmonization of public service provision and fortifying the redistributive potency of fiscal outlays. Corruption undermines the efficiency of fiscal spending intended to improve livelihoods and reduces the positive impact of public expenditure efficiency [36, 37]. The main reason for this is that corruption tends to allocate financial resources towards reducing expenditures on public services while increasing spending on public purchases and investments [38], leading to waste and inefficiency. Conversely, information and communication technologies offer a more convenient method to combat corruption by facilitating the establishment of anti-corruption systems and enabling more effective monitoring of fiscal fund utilization [39]. The fusion of the digital economy and finance not only helps curb rent-seeking behavior and corruption in fiscal revenue and expenditure but also enhances the accuracy of public spending. Additionally, electronic payment methods improve payment efficiency, reduce transaction time costs, and minimize expenses. Furthermore, the development of big data fosters improvements in government performance and effectively combats corruption [40].

Based on the analysis above, the digital economy potentially enhances the adaptive capacity of fiscal expenditure. However, due to the uncertain impact on income distribution and the possibility of non-linear effects, the adaptive capacity of fiscal expenditure influenced by the digital economy may also exhibit non-linear characteristics. Consequently, this study puts forth a hypothesis.

**Hypothesis 2**: **The advancement of the digital economy enhances the capacity of fiscal expenditure to adjust the income gap, with the existence of a non-linear correlation between the digital economy and the moderate capacity of fiscal expenditure**.

## Empirical research design

### Model setting

To empirically examine the influence of local fiscal expenditure on the regulation of intra-regional residential income disparity, we employ a two-way fixed-effect model:

$$Y_{it}=\beta_{0}+\beta_{1}Fisex_{it}+\beta_{c}X_{it}+\alpha_{i}+\mu_{t}+\varepsilon_{it}$$ (1)

Building upon Eq (1), this study incorporates the level of the digital economy and its interaction term with fiscal expenditure to investigate the moderating effect of the digital economy. The constructed model is as follows:

$$Y_{it}=\beta_{0}+\beta_{1}Fisex_{it}+\beta_{2}Dig_{it}+\beta_{3}Fisex_{it}\times Dig_{it}+\beta_{4}Dig_{it}^{2}+\beta_{5}Fisex_{it}\times Dig_{it}^{2}+\beta_{c}X_{it}+\alpha_{i}+\mu_{t}+\varepsilon_{it}$$ (2)

In the two equations provided above, the variable *Y*_*it*_ denotes the income disparity of region i in year t. The key explanatory variable *Fisex*_*it*_ corresponds to the fiscal expenditure level in region i during year t. The moderating variable *Dig*_*it*_ represents the level of the digital economy in region i during year t. The interaction term *Fisex*_*it*_ × *Dig*_*it*_ captures the joint influence of digital economy development and fiscal expenditure level. $Dig_{it}^{2}$ denotes the square of the digital economy’s development level, and $Fisex_{it}\times Dig_{it}^{2}$ represents the interaction term between fiscal expenditure and the squared term of the digital economy. This interaction term is utilized to reflect the non-linear influence of the digital economy on the adaptive capacity of fiscal expenditure. The control variables are denoted as *X*_*it*_. The regional fixed effects are represented by *α*_*i*_, the time-fixed effect is denoted as *μ*_*t*_, and the random disturbance term is symbolized as *ε*_*it*_.

#### Main variables and definitions

*Dependent variable*. Income disparity. This study considers the influence of demographic factors on regional income disparity within each region. The Theil index is employed as a quantitative measure to quantify income inequality. While previous studies have primarily used the Theil index to assess the urban-rural income gap [41], the current study focuses on examining income disparity within regions. Therefore, we measure the income disparity within each region by utilizing income and population data from the districts and counties within each region. This approach acts as a proxy variable for the income gap. The Thiel index is calculated as follows:

$$Theil_{it}=\sum_{j=1}^{n}\left(\frac{I_{ij,t}}{I_{i,t}}\right)\times ln\left(\frac{I_{ij,t}}{I_{i,t}}/\frac{P_{ij,t}}{P_{i,t}}\right)$$ (3)

In Eq (3), the variable i represents a specific region, while j ranges from 1 to n, indicating the districts and counties within the jurisdiction of region i. The term *I*_*ij*,*t*_ represents the total income of residents in district j under region i during period t, while *I*_*i*,*t*_ represents the total income of residents in region i during period t. Furthermore, *P*_*ij*,*t*_ denotes the total population of district j under region i during period t, while *P*_*i*,*t*_ signifies the overall population of region i during period t.

*Key explanatory variable*. Fiscal expenditure level. We use per capita general public budget expenditure as a metric to gauge the extent of local fiscal expenditure. Specifically, the fiscal expenditure level is determined by dividing the local fiscal expenditure by the total population of the region at the year-end.

*Moderating variable*. The level of the digital economy in each region. According to Zhao (2020) [42], the assessment of digital economy development considers multiple dimensions. These dimensions include the Internet penetration rate, which is determined as the ratio of Internet users to the population; the proportion of employees in computer services and software, serving as an indicator of the presence of Internet-related occupations; the level of Internet-related output, measured by per capita telecommunication services; the prevalence of mobile Internet usage, measured by the ratio of cell phone users to the population; and the progress in digital financial inclusion, evaluated using the China Digital Financial Inclusion Index. By employing a comprehensive evaluation system, these five sub-indicators are subjected to principal component analysis to derive an overall index that represents the overall development level of the digital economy.

*Control variables*. Various factors directly influence income inequality and distribution among regions. Firstly, the level of economic development among regions exerts a crucial influence on determining income inequality. Secondly, the extent of openness to global trade and investment affects residents’ income distribution through its impact on trade activities. Thirdly, the level of education and employment impacts income distribution by influencing both the accumulation of human capital and the availability of employment opportunities for residents. Fourthly, taxation, as a crucial instrument for secondary income redistribution, directly impacts residents’ income distribution. In addition, gross fixed capital formation in each region may also affect the income gap. Consequently, accounting for these variables in subsequent empirical analyses is imperative. According to the above analysis, the primary control variables in this study include: (1) Economic development level, represented by the per capita GDP in each region. (2) Level of external openness, quantified by the ratio of foreign direct investment to GDP. (3) Education level, measured by the proportion of students enrolled in general higher education institutions relative to the year-end resident population of the region. (4) Employment, assessed by the registered urban unemployment rate. (5) Tax revenue, indicating the proportion of tax revenue to local fiscal revenue, serving to control the impact of taxation on income distribution. (6) Gross fixed capital formation, expressed in terms of road kilometers.

*Instrumental variables*. In this paper, we employ the distance from prefecture-level cities to provincial capitals and the slope of prefecture-level cities as instrumental variables to address potential endogeneity issues in fiscal expenditure. The geographical distance refers to the distance between the prefecture-level city and the provincial capital city. The topographic slope is measured by calculating the average slope of each prefecture-level city.

### Data and descriptive statistics

The main dataset utilized in this research comprises panel data extracted from 276 regions across China, spanning the period from 2011 to 2020. The income and population data necessary for calculating the Thiel index, pertaining to the districts and counties within each municipality, are primarily sourced from the statistical yearbooks of provinces and cities, as well as the statistical bulletins of individual districts and counties.

The evaluation of digital economy development predominantly relies on data extracted from the statistical yearbooks of respective regions, along with the China Digital Financial Inclusion Index published by Peking University. Geographical distance is primarily measured based on the latitude and longitude coordinates of each city. The calculation of topographic slope relies on data from ASTER Global Digital Elevation Model V003, and ArcGIS software is used for this purpose. As for the remaining variables, data primarily originate from the statistical yearbooks and statistical bulletins of each city.

Data selection and exclusion procedures were implemented to safeguard the integrity and reliability of the dataset. Firstly, cities within the Tibet Autonomous Region were excluded from the sample due to a significant number of missing values in their data. Secondly, Hainan Province was also excluded from the analysis due to its adoption of a three-level government structure, where prefecture-level cities usually do not govern counties and county-level cities.

For detailed information regarding variable definitions, calculation methods, and descriptive statistics, please refer to Table 1.

**Table 1:** Variable definition and descriptive statistics.

Variable Name	Variable Definition	Number of observations	Mean value	Standard deviation	Minimum value	Maximum value
Theil	Thiel’s index	2760	0.0831	0.0433	0	0.2564
Lnfisex	Fiscal expenditure per capita is taken as the logarithm	2760	8.9968	0.5256	6.3443	11.6025
Dig	Comprehensive Digital Economy Development Index by Region	2760	-0.0238	0.4696	-0.5695	9.901
Lndis	The distance from each regional center city to the provincial capital city is taken as the logarithm	2460	5.0329	0.6746	3.0956	7.059
Slope	The average slope of each region	2760	10.8192	5.6262	1.5921	27.1388
Lngdp	GDP per capita by region is taken as the logarithm	2760	10.6954	0.5677	8.7729	13.0556
Copen	Foreign direct investment amount as a percentage of GDP	2760	1.6478	2.0601	0	56.94
Edu	Number of students enrolled in higher education as a percentage of the total population	2760	1.8252	2.358	0	14.64
Unemp	Urban registered unemployment rate	2760	3.0776	0.741	1.05	8.3
Fis1	Share of tax revenue in fiscal revenue	2671	70.2534	10.6286	20.28	100
Lnfixcap	The number of road kilometers follows a logarithmic scale	2760	9.2886	0.7372	4.9081	12.1051

## Empirical results

### Benchmark regression

The primary objective of the baseline regression analysis is to assess the influence of the magnitude of fiscal expenditure on income inequality. To account for the potential influence of time-invariant and individual-invariant factors, a two-way fixed-effects model (FE) is utilized in this study. Additionally, robust standard errors with regional clustering are utilized in the estimation process to account for potential heteroskedasticity, cross-sectional correlation, and time series correlation. The findings of the benchmark regression analysis, which investigates the connection between fiscal expenditure size and regional income inequality, are presented in Table 2. Columns (1) and (2) show the results of ordinary least squares (OLS) regression, both without and with the inclusion of control variables, respectively. The results reveal a significant negative correlation between fiscal expenditure and income disparity, suggesting that fiscal expenditure plays a significant role in reducing income disparity across China’s regions. Columns (3) and (4) present the outcomes of the fixed-effects regression analysis, incorporating individual fixed effects and the combination of individual and time fixed effects (FE). Even after controlling for time-invariant and individual-invariant factors, the coefficients associated with fiscal expenditure size remain negative and statistically significant. These findings suggest that an increase in fiscal spending levels contributes to the reduction of the income gap, which partially supports research hypothesis 1.

**Table 2:** Benchmark regression results for the effect of fiscal expenditure size on income disparity.

Variables	OLS	OLS	FE	FE
	(1)	(2)	(3)	(4)
Lnfisex	-0.041*** (0.002)	-0.032*** (0.003)	-0.036*** (0.004)	-0.017*** (0.004)
Control Variables	NO	YES	YES	YES
City Fixed	NO	NO	YES	YES
Year Fixed	NO	NO	NO	YES
Observations	2760	2671	2671	2671
R-squared	0.5104	0.5255	0.527	0.5919

Note:

*** and ** denote 1% and 5% significance levels, respectively; robust standard errors for clustering to cities are in parentheses. Same as below.

### The moderating effects

The preceding theoretical analysis establishes the potential impact of digital economy development on the efficacy of fiscal expenditure in addressing income inequality. To further investigate this phenomenon, this section integrates the level of digital economy development and its interactive relationship with fiscal expenditure into the model, to examine the moderate effect of the digital economy. Table 3, column (1), presents the regression results of the two-way fixed-effect model, including digital economy variables. The results demonstrate a significant negative correlation between fiscal expenditure and the income gap, whereas the coefficient between the digital economy and the income gap does not attain significance. Additionally, column (2) presents the regression results that incorporate the square term of the digital economy, confirming the U-shaped impact of the digital economy on the income gap. This suggests that the digital economy initially contributes to narrowing the income gap, but beyond a certain threshold, it exacerbates the income gap. Column (3) displays the regression results subsequent to the inclusion of the interaction between digital economy development and fiscal expenditure. The U-shaped effect of the digital economy on the income gap persists, and the coefficient of the interaction term between the digital economy and fiscal expenditure is positive, indicating that the digital economy may initially diminish the adjustment ability of fiscal expenditure. However, The interaction coefficient between the square term of the digital economy and financial expenditure becomes negative, suggesting that as the digital economy develops, it strengthens the effect of fiscal expenditure in narrowing the income gap and enhances the income redistribution adjustment ability of fiscal expenditure.

**Table 3:** Moderating effects of the digital economy on the size of fiscal expenditures.

Variables	theil			
	(1)	(2)	(3)	(4)
Lnfisex	-0.017*** (0.004)	-0.017*** (0.004)	-0.016*** (0.004)	-0.014*** (0.004)
Dig	-0.02 (0.002)	-0.009*** (0.002)	-0.073*** (0.025)	-0.142*** (0.028)
Dig^2^		0.001*** (0.000)		0.04*** (0.009)
Lnfisex × Dig			0.008*** (0.003)	0.014*** (0.003)
Lnfisex × Dig^2^				-0.004*** (0.001)
Control Variables	YES	YES	YES	YES
City Fixed	YES	YES	YES	YES
Year Fixed	YES	YES	YES	YES
Observations	2671	2671	2671	2671
R-squared	0.5929	0.5960	0.5972	0.6053

These findings confirm the second research hypothesis posited in this paper, which states that the digital economy augments the income adjustment ability of fiscal expenditure with non-linear effects.

### Endogeneity issues

In order to address potential endogeneity issues resulting from the reciprocal relationship between income disparity and fiscal spending, this study adopts the instrumental variables approach to estimate the fixed effects model. Neighboring regions not only share geographic proximity but also have stronger economic interdependencies, leading to the generation of economic spillover effects. Proximity to provincial capital cities is associated with stronger incentives for regions to improve infrastructure development, offer better public services, and consequently increase fiscal expenditures. Furthermore, the financial allocation for enhancing transportation infrastructure in different districts represents a significant component of local government expenditure on public services, indicating the policy emphasis in the regional fiscal budget. Additionally, if a region has favorable initial topographic conditions for infrastructure construction and population concentration, it may benefit from advantages and greater efficiency in fiscal spending during urbanization initiatives. Therefore, this paper chooses the spherical distance from each prefecture-level city to the provincial capital city and the slope of each prefecture-level city as instrumental variables to address potential endogeneity concerns.

Considering the time-invariant characteristics of geographic distance and topographic slope, this study follows a similar method to Nunn and Qian (2014) in addressing the use of two-dimensional instrumental variables [43]. Specifically, the two variables, namely the geographic distance and topographic slope between each prefecture-level city and its corresponding provincial capital city, are incorporated into the model along with the cross-product term of the national mean per capita fiscal expenditure, which exhibits variation across individuals. These variables are utilized as instrumental variables for fiscal expenditures, aiming to address the issue of data dimensionality associated with cross-sectional instrumental variables.

The findings obtained using the instrumental variables method are presented in Table 4. Columns (1) and (2) display the regression outcomes that consider only the instrumental variable for fiscal spending. These results align with the findings from the baseline regression and reveal a significant negative correlation between fiscal expenditure and the income gap. They indicate that an increase in fiscal expenditure helps narrow the income gap. Columns (3) and (4) exhibit the regression outcomes that take into account the adjustment effect of the digital economy. Compared to Table 3, the results show no significant change. Both the coefficients of fiscal expenditure and the digital economy are significantly negative, indicating a favorable impact on narrowing the income gap. Furthermore, the interaction coefficient between fiscal expenditure and the digital economy is positive, while the interaction coefficient between fiscal expenditure and the square of the digital economy is negative. These findings indicate that, under the instrumental variable method, the development of the digital economy continues to have a non-linear impact on the adjustment ability of fiscal expenditure regarding the income gap. Additionally, the p-value of the Kleibergen-Paap rk LM statistic is less than 0.01 in the instrumental variable unidentifiable test, leading to the rejection of the null hypothesis of "under-identified instrumental variables" at a significance level of 1%. Furthermore, in the weak instrumental variable test, the Wald F-statistic of Kleibergen-Paap rk surpasses the critical value at the 10% level of the Stock-Yogo weak identification test, confirming the suitability of the instrumental variable selection.

**Table 4:** Regression results of instrumental variables.

Variables	(1)	(2)	(3)	(4)
Lnfisex	-0.048*** (0.001)	-0.052*** (0.003)	-0.039*** (0.003)	-0.039*** (0.004)
Dig			-0.265*** (0.057)	-0.292*** (0.056)
Dig^2^			0.030* (0.016)	0.038** (0.053)
Lnfisex × Dig			0.029*** (0.006)	0.031*** (0.006)
Lnfisex × Dig^2^			-0.003** (0.002)	-0.004** (0.002)
Control Variables	NO	YES	NO	YES
Kleibergen-Paap rk LM statistics	943.077 [0.0000]	315.358 [0.0000]	92.460 [0.0068]	102.461 [0.0003]
Kleibergen-Paap rk Wald F statistic	2625.817 {19.93}	417.944 {19.93}	25.259 {6.61}	30.416 {6.61}
Observations	2460	2380	2460	2380
R-squared	0.5305	0.5348	0.5373	0.5482

Note: [] denotes P-values, and {} represents critical values for the 10% level of the Stock-Yogo weak identification test.

### Robustness tests

This paper conducts several robustness tests to ensure the robustness of the study findings and mitigate potential biases arising from sample selection and indicator choice. Firstly, the study uses the urban-rural income gap as the explanatory variable and employs the urban-rural Theil index, as suggested by Xie (2021) [41], to measure the income gap within each region. Secondly, the study modifies the measure of fiscal expenditure level by using the ratio of local fiscal expenditure to local GDP (Fisex). Thirdly, the study utilizes the entropy method to assess the level of the digital economy (Digit). Lastly, Beijing, Tianjin, Shanghai, and Chongqing are excluded from the analysis due to the significant disparities between municipalities and prefecture-level cities. The outcomes of the robustness tests are presented in Table 5. The regression results in columns (1) and (2) using the alternative explanatory variables indicate that the level of fiscal expenditure is still significantly and negatively related to the income gap, while the moderating effect of the digital economy continues. The results in columns (3) and (4) show the outcomes after modifying the measures of fiscal expenditure and the digital economy, with the coefficient of fiscal expenditure slightly surpassing that of the baseline regression. Importantly, even after altering the measurement method of the digital economy, it continues to exert a non-linear impact on the moderate capacity of the income gap in fiscal expenditure. In columns (5) to (6), the regression results are presented after eliminating specific samples, and they consistently validate the findings of the reference regression. The results obtained from these robustness tests demonstrate minimal deviations compared to the baseline regression, highlighting the robustness of the findings presented in this paper.

**Table 5:** Robustness test results.

Variables	Substitution of explanatory variables		Change the measurement of variables		Excluding some samples	
	(1)	(2)	(3)	(4)	(5)	(6)
Lnfisex	-0.015*** (0.005)	-0.012*** (0.004)		-0.019*** (0.004)	-0.018*** (0.004)	-0.014*** (0.004)
Dig(Digit)		-0.024*** (0.027)		-0.936*** (0.339)		-0.130*** (0.029)
Dig^2^(Digit^2^)		0.010** (0.004)		2.558*** (0.733)		0.039*** (0.010)
Fisex			-0.077*** (0.019)			
Lnfisex × Dig		0.103*** (0.027)		0.088** (0.035)		0.130*** (0.003)
Lnfisex × Dig^2^		-0.066** (0.033)		-0.257*** (0.077)		-0.004*** (0.001)
Control Variables	YES	YES	YES	YES	YES	YES
Observations	2671	2671	2671	2671	2631	2631
R-squared	0.0683	0.0762	0.5912	0.6002	0.5947	0.6065

### Further analysis

The previous section analyzed the impact of the size of fiscal expenditure on income disparity and its interaction with the digital economy. In this section, we redirect our attention to the impact of different types of fiscal expenditures on income disparity, taking into account the structure of fiscal expenditure. Furthermore, we explore whether the digital economy has strengthened the moderating effect of disaggregated fiscal expenditures on income disparity.

#### The influence of categorized fiscal expenditure on the income gap

We examine the impact of individual fiscal expenditure categories on income disparity. Acknowledging that different types of fiscal expenditures have diverse effects, this study primarily concentrates on analyzing the effects of particular expenditure categories on income disparity. Specifically, we examine the impact of social security and employment expenditures (Fisex1), education expenditures (Fisex2), health expenditures (Fisex3), and expenditures related to agriculture, forestry, water, and urban and rural affairs (Fisex4), which are recognized for their substantial roles in regulating income distribution.

Based on the results of the two-way fixed effects regressions presented in Table 6, several observations can be made: (1) The coefficient of social security expenditure is positive but not statistically significant, indicating that local fiscal social security expenditure does not effectively reduce the income gap. This can be attributed to two reasons. Firstly, there are significant variations in the coverage of social security expenditures, with higher coverage rates observed among urban groups compared to the rural population. Moreover, there are notable differences in social security coverage rates across various occupational groups. Secondly, social security expenditures are primarily concentrated on two items: pensions for administrative and institutional units and pensions for urban enterprise employees, with a relatively low proportion allocated to other expenditures. This situation increases the income gap among the retired and limits social security’s income-distribution effects.

**Table 6:** Baseline regression results for the impact of categorized fiscal expenditure on income disparity.

Variables	theil			
	(1)	(2)	(3)	(4)
Fisex1	0.025 (0.021)			
Fisex2		0.083*** (0.024)		
Fisex3			0.135*** (0.031)	
Fisex4				-0.019** (0.009)
Control Variables	YES	YES	YES	YES
City Fixed	YES	YES	YES	YES
Year Fixed	YES	YES	YES	YES
Observations	2584	2670	2548	2293
R-squared	0.5694	0.5832	0.5756	0.4986

(2) Both education expenditure and health expenditure are significantly positively correlated with the income gap, suggesting that they contribute to the widening of the income gap. Today’s society relies heavily on education to promote income mobility and intergenerational class mobility. It serves as the primary mechanism for individuals to improve their social standing, overcome socioeconomic barriers, and reduce income inequality. However, several factors impede the mitigating effect of education expenditures on income disparities. Firstly, there are significant disparities in the distribution of basic education resources, such as teachers, school facilities, and teaching expenses, between urban and rural areas, leading to substantial inequalities in higher education opportunities between these regions. As a result, education expenditures have not effectively promoted equal educational opportunities, thereby limiting their impact on reducing income disparities. Secondly, the unequal distribution of education resources may perpetuate the "Matthew effect" in education, thereby perpetuating the existing unequal social class structure. From the perspective of health expenditure, China’s health spending continues to favor urban areas, with significantly greater medical resources compared to rural areas. Furthermore, China’s public health expenditure has a limited impact on the health of residents in rural areas and the central and western regions, thereby reducing the effectiveness of health expenditure in moderating health disparities and potentially widening the income gap.

(3) The coefficients of agriculture, forestry, and water expenditure, as well as urban-rural affairs expenditure, show a significantly negative association with the income gap, indicating that these expenditures help narrow the income gap. The allocation of funds to agriculture, forestry, and water expenditure, as well as urban-rural affairs expenditure, demonstrates a strong inclination towards rural areas. This allocation facilitates infrastructure development between urban and rural regions, contributes to the advancement of rural undertakings, promotes the reduction of the income gap between these areas, and consequently reduces income disparity within the region.

In conclusion, considering the prevailing urban-rural gap, the study indicates that fiscal expenditures with an urban-biased, including social security, education, and health, face challenges in effectively regulating the income gap and may contribute to its widening. Conversely, fiscal expenditures with a greater rural bias, such as those allocated to agriculture, forestry, water, and urban-rural affairs, play a crucial role in reducing the income gap. These findings also partially support research hypothesis 1 that the adjustment ability of different types of fiscal expenditure varies.

#### The moderating effect of the digital economy on categorized fiscal expenditure

This section tests the moderating effect of the digital economy on categorized fiscal expenditure. Table 7 displays the regression results that examine how the digital economy moderates different types of fiscal expenditure. Columns (1)-(2) present the regression outcomes indicating the moderating effect of the digital economy on social security expenditure. After accounting for the moderating effect of the digital economy, the persistence of the U-shaped relationship between the digital economy and the income gap becomes evident. However, the correlation between social security expenditure and the income gap remains nonsignificant, as does the interaction term between the digital economy and social security expenditure. Columns (3)-(4) display the regression results that examine how the digital economy influences the adjustment ability of education expenditure on the income gap. When the digital economy is considered, education expenditure remains significantly positively correlated with the income gap. The coefficient of the interaction term between education expenditure and the digital economy is negative, whereas the interaction term between education expenditure and the square of the digital economy is positive. These findings indicate that the initial effect of the digital economy is to weaken the influence of education expenditure on widening the income gap. However, as the digital economy further develops, it strengthens the positive relationship between education expenditure and the income gap. Columns (5)-(6) display the regression results that examine how the digital economy regulates the impact of health service expenditure on the income gap. The coefficient of the interaction term between health service expenditure and the square of the digital economy is significantly positive. This indicates that when health service expenditure has the potential to widen the income gap, the digital economy further amplifies the "widening" effect. The results in columns (7)-(8) indicate that the coefficient of expenditure on agriculture, forestry, and water resources is significantly negative. The interaction term between the digital economy and this expenditure is significantly positive, whereas the interaction term between the square of the digital economy and this expenditure is negative. These findings suggest that as the level of the digital economy improves, there is an initial weakening effect on the income redistribution capacity of agriculture, forestry, and water expenditure, as well as urban and rural affairs expenditure. However, beyond a certain threshold, the digital economy contributes to enhancing the income redistribution ability of these expenditures.

**Table 7:** Moderating effects of the digital economy on the structure of fiscal expenditure.

Variables	theil							
	(1)	(2)	(3)	(4)	(5)	(6)	(7)	(8)
Fisex1	0.019 (0.016)	0.016 (0.016)						
Fisex2			0.083*** (0.023)	0.065*** (0.024)				
Fisex3					0.146*** (0.031)	0.130*** (0.034)		
Fisex4							-0.017** (0.008)	-0.018*** (0.006)
Dig	-0.010*** (0.003)	-0.014*** (0.003)	-0.010*** (0.002)	0.009 (0.009)	-0.012*** (0.002)	-0.010** (0.005)	-0.013*** (0.003)	-0.025*** (0.003)
Dig^2^	0.001*** (0.000)	0.003 (0.000)	0.001*** (0.000)	-0.007** (0.004)	0.001*** (0.000)	-0.001 (0.002)	0.001*** (0.000)	0.004*** (0.001)
Fisex_i_ ×Dig		0.029 (0.025)		-0.105** (0.044)		-0.028 (0.043)		0.058*** (0.014)
Fisex_i_ ×Dig^2^		-0.010 (0.014)		0.046** (0.023)		0.032* (0.019)		-0.012* (0.007)
Control Variables	YES	YES	YES	YES	YES	YES	YES	YES
City Fixed	YES	YES	YES	YES	YES	YES	YES	YES
Year Fixed	YES	YES	YES	YES	YES	YES	YES	YES
Observations	2609	2609	2671	2671	2548	2548	2293	2293
R-squared	0.5806	0.5811	0.5883	0.5910	0.5821	0.5829	0.5103	0.4816

In comparing the moderating effects of the digital economy on different types of fiscal expenditure, it is apparent that the moderating effect on education expenditure is the most significant, followed by agriculture, forestry, and water resources expenditure, as well as urban and rural affairs expenditure. The moderating effect on health expenditure is the weakest, and there is no significant moderating effect on social security expenditure.

## Discussion

Our findings are based on panel data from 276 cities in China spanning the years 2011 to 2020. The research demonstrates that overall fiscal expenditure contributes to narrowing the income distribution gap. Additionally, the digital economy enhances the income adjustment ability of fiscal expenditure with non-linear effects. This finding remains robust after conducting a series of tests.

Previous studies have investigated the impact of fiscal expenditure on income distribution [1–7]. However, there is a research gap in understanding the dynamics of income distribution effects within the context of the digital economy, as well as the variations in the redistributive role of different types of fiscal expenditure. With the development of the digital economy, its impact extends across all facets of the economy, emphasizing the necessity of incorporating it into the analytical framework. In contrast to previous research, our study accounts for the moderating effect of the digital economy. We employ fixed effects models and instrumental variable approaches to investigate the moderating effect of the digital economy on the income redistributive ability of fiscal expenditure, as well as the differential effects of the digital economy on various types of fiscal expenditure. Furthermore, a series of robustness tests will be conducted to ensure the reliability of our conclusions.

The present study provides support for and expands upon previous findings in several aspects. Consistent with prior research [4–11], our findings indicate that fiscal expenditure reduces the income distribution gap. However, local fiscal social security was not effective in narrowing the income distribution gap, while education expenditure and health expenditure had a reversed impact. On the other hand, expenditure on agriculture, forestry, water resources, and urban and rural affairs contributed to narrowing the income distribution gap. Additionally, we investigate how various types of fiscal expenditure moderate income distribution in the digital economy context. Our findings reveal that the digital economy has no significant moderating effect on social security, while it has a U shape moderating effect on education expenditures and exacerbates the impact of health expenditure in widening the income distribution gap. The digital economy only positively amplifies the income redistribution capacity of expenditure on agriculture, forestry, water resources, and urban and rural affairs. Consequently, our conclusion suggests that rural-biased fiscal expenditure, specifically expenditure on agriculture, forestry, and water resources, plays a more pronounced role in income distribution adjustment, whereas expenditure on social security, education, and health services yields negligible effects. In subsequent economic development, it is imperative to maintain focus on expenditure in this domain and fully leverage the positive impact of private fiscal expenditure in reducing the income distribution gap.

The present analysis has several noteworthy limitations to consider. Similar to previous literature, our assessment is susceptible to unmeasured confounding factors that may not be adequately accounted for by robust research-specific adjustments and statistical analysis methods. Furthermore, due to missing data for certain cities, it was not possible to include all cities in China in the analysis. Lastly, the substantial amount of missing data regarding fiscal expenditure classification limits our ability to discuss the adjustment effect of different types of fiscal expenditure on the income gap in greater detail.

Owing to the intricate connotation and extensive scope of the digital economy, coupled with the limited availability of statistical information, constructing an indicator system that comprehensively captures the level of digital economy development remains highly challenging. Moving forward, we will enhance the following aspects: Firstly, we will persist in researching the digital economy and refine the indicator system in alignment with regional development circumstances. Secondly, we will expand our investigation to the county level and delve into the disparities arising from variations in digital economy development across different regions. Lastly, we will intensify our study on the impact mechanism of the digital economy, conducting in-depth analyses of heterogeneity, driving factors, and critical pathways influencing the digital economy’s effects in diverse regions.

## Conclusions and recommendations

The digital economy has emerged as a new driver of economic development, while fiscal expenditure, as a crucial tool for fiscal policy regulation, plays a pivotal role in addressing income inequality. This study integrates these two factors within a unified analytical framework. By employing a combination of theoretical analysis and empirical testing using regional panel data from 2011 to 2020, the research investigates the impact of the fiscal expenditure scale on regional income disparity. Additionally, the study explores the influence of different types of fiscal expenditure structures on the income gap.

The key findings of this investigation encompass three fundamental aspects. Firstly, it is established that overall fiscal expenditures exhibit a significant and negative relationship with the income gap. In other words, an increase in the level of fiscal expenditures contributes to the narrowing of regional income disparity. Secondly, significant disparities exist in the effectiveness of different types of fiscal expenditure in addressing the income gap. Social security expenditure, education expenditure, and health expenditure, which exhibit a strong urban bias, have not been successful in significantly narrowing the income gap. In fact, they may even contribute to its widening. On the other hand, agriculture, forestry, and water expenditure, along with urban and rural affairs expenditure, play a crucial role in reducing the income gap. Thirdly, the development of the digital economy exhibits a U-shaped effect on the income gap. Initially, it contributes to narrowing the income gap. However, beyond a certain threshold, it has the opposite effect, widening the income gap. Fourthly, the digital economy has a non-linear impact on the income gap adjustment ability of fiscal expenditure. It initially weakens the adjustment ability and then strengthens it. From a categorized perspective, the digital economy primarily enhances the moderating effect of fiscal expenditure on agriculture, forestry, and water resources, contributing to narrowing the income gap. However, it intensifies the impact of education expenditure and health expenditures in widening the income gap.

Based on the aforementioned findings, this study highlights the superior effectiveness of fiscal expenditures with a rural bias, such as those allocated to agriculture, forestry, and water resources, in regulating income disparity compared to expenditures on social security, education, and health. These findings have important implications for future governmental decision-making, and the following considerations should be taken into account:

Firstly, it is recommended to maintain an appropriate increase in total fiscal expenditure. This will leverage the regulatory effect of fiscal expenditure on regional income distribution gaps. Additionally, optimizing the structure of central and local fiscal expenditures is necessary. Specifically, the focus should be on regulating fiscal expenditures related to livelihood to reduce urban bias. This involves increasing social security provisions and coverage in rural areas, as well as tailoring livelihood-focused fiscal investments to local conditions. These actions will contribute to narrowing the urban-rural and regional income gaps.

Secondly, to fully leverage the regulatory role of the digital economy, it is necessary to promote the integration of the digital economy and government governance. The digital transformation in the fiscal sector should be deepened to enhance fiscal transparency and expenditure efficiency, thereby amplifying its redistributive moderate capacity. Efforts should also be made to advance Internet infrastructure development and improve the accessibility and universality of Internet knowledge. This will help reduce the digital divide among different groups and segments, enabling a swift transition to digital dividends and strengthening the adjustment capacity of fiscal expenditure. Lastly, while prioritizing high-quality economic development, it is crucial to ensure fairness and justice and strike a balance between economic growth and common prosperity. This requires expanding the "cake" while narrowing the income gap and ensuring an equitable distribution of resources, ultimately achieving shared prosperity.

Thirdly, we should deepen the reform of the fiscal system by defining the financial and administrative powers of the central and local governments, improving their relationship, increasing transfer payments from the central government to local governments, enhancing general transfer payments, rectifying special transfer payments, and aligning the financial resources, expenditure responsibilities, and powers of local governments to meet their public service needs. We should also accelerate substantive reform of the local fiscal system by decentralizing local fiscal power, reforming the tax distribution system below the provincial level, enhancing the local tax system, expanding local financial sources, strengthening oversight of local fiscal budgets and expenditure management, managing local fiscal risks, and improving the performance management of local fiscal expenditures.

## Supporting information

S1 Dataset (DTA)
